# A different approach to teaching pre-clerkship students physical diagnosis: standardized patient instructor-senior medical student teaching teams

**DOI:** 10.1186/s12909-023-04782-4

**Published:** 2023-11-21

**Authors:** Audrey Spelde, Benjamin Blatt, Karen L. Lewis, Jennifer L. Owens, Larrie Greenberg

**Affiliations:** The George Washington University School of Medicine and Health Sciences, 9109 Fall River Ln, Potomac, Md 20854 USA

**Keywords:** Physical diagnosis, Co-teaching, Medical students as teachers, Standardized patients, Physical exam, Pre-clerkship instruction

## Abstract

**Background:**

Faculty have traditionally taught the physical examination (PE) to novice medical students (pre-clerkship students.), despite recruiting and cost issues and problems standardizing their approach.

**Activity:**

We present a model using standardized patient instructor (SPI)-fourth year medical student (MS4) teams to teach PE to pre-clerkship students, leveraging the benefits of co-teaching and peer-assisted learning.

**Results:**

Surveys of pre-clerkship students, MS4s and SPIs indicate positive perceptions of the program, including MS4s reporting significant growth in their professional identities as educators. Pre-clerkship students' performance on the spring clinical skills exams was equivalent to or better than their peer performance pre-program implementation.

**Implications:**

SPI-MS4 teams can effectively teach novice students the mechanics and clinical context of the beginners’ physical exam.

**Supplementary Information:**

The online version contains supplementary material available at 10.1186/s12909-023-04782-4.

## Background

Novice medical students (pre-clerkship students) learn basic physical exam (PE) at the start of medical school. Teaching the PE to pre-clerkship students. has been traditionally assumed by faculty; however, using faculty has downsides in that faculty1) are expensive; 2) are difficult to recruit; 3) receive little credit toward academic promotion; 4) are difficult to standardize; 5) may miss subtle and obvious mistakes by trainees [1, 2]; 6) may be unfamiliar with evidence-based teaching techniques [3]; and 7) may feel inadequate in teaching the PE [4].

**Fig. 1:**
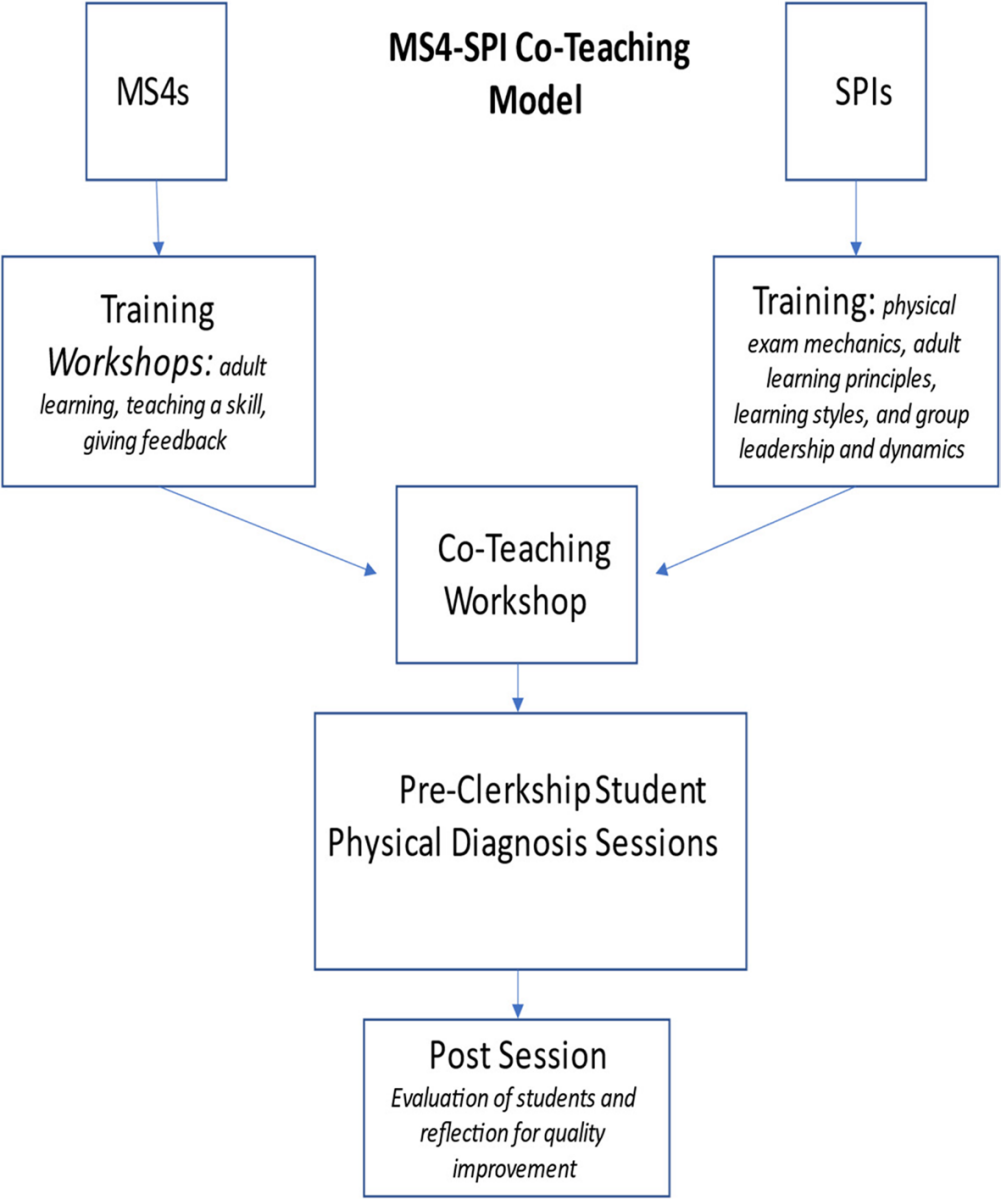
Summary of MS4-SPI Co-Teaching Model. MS4: fourth-year medical students; SPI: standardized patient instructor

Successful PE training models have been developed using real patients [5], senior level medical students or residents [6, 7], and lay people [8] as teachers. Importantly, what is common to all of these models is that student performance on the PE has not declined by precluding faculty involvement [5, 7]. Lay educators, however, lack expertise in clinical context [9] --essential for students to test diagnostic hypotheses using PE data. To address the need for standardization and clinical context in teaching the PE, one group of educators added hypothesis-driven diagnostic exercises to their lay teaching [10]. At George Washington University School of Medicine (GWU), we have addressed this need with a model featuring standardized patient instructor (SPI)-senior medical student (MS4) teams. (Fig. 1) SPIs are paired with MS4s to teach PE to pre-clerkship students. SPIs offer expertise in exam mechanics; MS4s, in clinical context. This model leverages the benefits of co-teaching, a powerful facilitator of learning [11]. Since virtually all US and many non-US medical schools use SPs [12, 13] and many have students-as-teachers programs, this model has the potential for wider application. The purpose of this paper is to  describe this unique SPI-MS4 team PE teaching model (Fig. 1).

### Activity

The required Physical Diagnosis (PDX) at GWU is one component of the Practice of Medicine pre-clerkship clinical skills course. The other components are 1) Clinical Integration (PBL-like small group sessions); 2) Interview; 3) Formative OSCE exercises; 4) Clinical Apprenticeship (applying clinical skills with a practicing physician) and 5) Professional Development Coaching. PDX is taught in groups of 4 -5 pre-clerkship students working with the same SPI-MS4 teams, meeting for 3 hours 6 times/year. Class size is ~ 180 students and sixty to ninety MS4s elect to be instructors in the PDX course every year.

MS4s receive teacher training through our TALKS (Teaching and Learning Knowledge and Skills) senior students-as-teachers elective, which provides workshops on adult learning principles, teaching a skill, and giving feedback [14]. SPIs undergo intensive training in a longitudinal program developed by the Assistant Director of our CLASS simulation center (JO). Structured around a faculty-developed manual, the SP curriculum includes adult learning principles, learning styles, and group leadership and dynamics. Specifically, SPI training and standardization occurs in multiple episodes, beginning in summer and lasting throughout the academic year. The curriculum includes how to teach, communicate, and facilitate; how the course fits into the rest of the curriculum; how to give feedback; how to do the PE maneuvers and teach them to the students. To assess competence to participate in the program, the SPIs are required to pass mastery tests conducted by the SP Educators.

MS4s and SPIs also participate together in a 2-hour team workshop to delineate their complimentary roles in planning and implementing their sessions and evaluating pre-clerkship students. The underlying workshop constructs are the GRPI model (Goals, Roles, Process and Interpersonal factors) and Mezirow’s transformational learning theory (process, premise and content) to impart concepts of interdisciplinary teaching (Suppement) [15, 16]. Working together as co-teachers is in keeping with social and experiential learning theory: learning is created in the social exchange among team members [17].

The PDX sessions, built around the Core and Clusters (C+C) model [18], teach PE over 18 months within the context of clinical reasoning, with each cluster session focused around a common patient presentation. Initially, students are taught the first component of C+C-- a~ 40 maneuver core exam that samples the major organ systems. The core exam is a streamlined and practical physical exam that is less cognitively burdensome than the traditional head-to-toe exam. The core exam is well-suited to prepare learners for early clinical experiences, which many schools are adopting. Students then advance to the second component of C+C--the diagnostic clusters, which are sets of hypothesis-driven H&P organized around specific, common clinical presentations, designed to develop clinical reasoning skills. Chest Pain is an example of such a clinical presentation (Table 1). Clusters draw basic maneuvers from the core exam (e.g., basic heart auscultation) and add additional, specialized maneuvers useful in discriminating among diagnostic possibilities (e.g., listening for extra heart sounds in the lateral decubitus position). C+C is taught over 18 months and the course is sequential in that students are initially taught the ~40 maneuver core exam, and then, thus prepared, move to the clusters, each of which demonstrates a clinical presentation representative of the organ system block the students are experiencing (e.g., the Chest Pain and Dyspnea Clusters occur during the CardioPulmonary Block) (Table 2).

**Table 1:** Example of a George Washington University School of Medicine MS1 cluster PDX session: chest pain

**Time**	**Content**
**Pre-Session**	1. Students read Chest Pain Cluster packet and review chapter 9 in Bates’ *Guide to Physical Examination* as needed (be prepared to answer questions about the items in this packet) *2.* Students watch Chest Pain Cluster video on Blackboard 3. Students prepare any questions regarding the material to be covered
4:30-5:15pm	**I. Introduction of Session** 1. Introduce the session – MS4s & SPIs 2. Session-specific Socratic Min-Quiz – MS4s 3. Review of Cluster Algorithm – MS4s 4. Open the floor for questions – MS4s & SPIs Clinical knowledge questions to be answered by MS4s; examination questions to be answered by SPI. SPI demonstrates on MS1 as needed.
5:15-5:45 pm	**II. Peer and SPI Practice of the Chest Pain Cluster** Students practice the Chest Pain Cluster Maneuvers on each other and SPIs in their groups. Each MS1 will be an examiner and examinee at least once. MS4s and SPIs observe and correct technique as needed.
5:45-7:15 pm	**III. Case Based Practice with SPIs, using Clinical Relevance cases:** In pairs, students will work through 3 Clinical Relevance cases together. The group will debrief with the PIs & SPI after each case.
7:15-7:30 pm	**IV. Wrap Up and Reflection** PIs and SPIs answer further questions. The group should also answer the session’s reflection/discussion questions aloud.
7:30-8	**SPI-MS4 Team Meeting** PIs and SPIs complete evaluation form on each student; share impressions of their co-teaching, identifying any changes they plan to make in their teaching collaboration for the next session

**Table 2:** Correlation of the core and clusters with the curriculum organ system blocks

**Core/Cluster**	**Organ System Block**
Core Sessions A,B,C,D	Foundations
Fever	Infectious Disease, Immunology
Dyspnea	Cardiopulmonary
Chest pain	Cardiopulmonary
Lower Extremity: Back, Hip, Knee	Musculoskeletal
Upper Extremity: Shoulder, Elbow, Wrist	Musculoskeletal
Neurology: Facial Weakness, Falls	Brain and Behavior

**Fig. 2:**
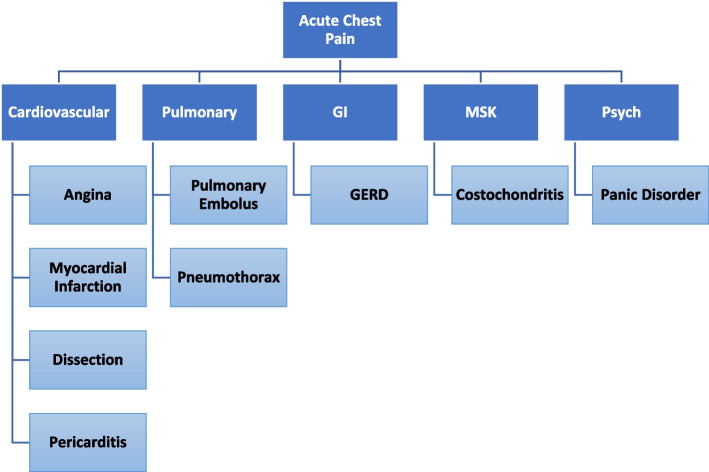
Chest pain schema

To prepare for the PDX sessions, pre-clerkship students review relevant diagnostic schema (Fig. 2) and PE in the PDX Manual, their physical diagnosis textbook, and illustrative videos. The total time for needed for students to prepare for the sessions is ~ 60-90 minutes. It includes reading the Cluster Packet (12 pages), reading a Bates chapter (~20 pages), and viewing the video (2-6 minutes) [19]. The MS4-SPI teams uniformly conduct the session using the format prescribed in the Manual (Table 1). They begin with an oral mini-quiz (usually 5-7 items) on pre-session knowledge (e.g., What is the physiology and significance of an S3? What diagnoses does its presence support in a patient presenting with shortness of breath?). They then review the diagnostic schema and address pre-clerkship students’ uncertainties. The rest of the session is graduated practice. First, the pre-clerkship students practice PE maneuvers on each other and on the SPIs, with team feedback. Finally, the SPIs present them with cases--“mini-formative OSCEs.” Working in pairs, students read a history and deduce discriminating maneuvers to perform on the SPI. Then, based on simulated physical findings, the pre-clerkship students develop hypotheses and present the most likely diagnosis. Post-session, the SPI-MS4 team evaluates each student, then the team self-assesses and identifies improvements for their next teaching session (Table 1). Feedback is a key element of the course. SPIs and MS4s give formative feedback “in the moment” throughout each session: 1) as students attempt the maneuvers on each other and on the SPIs; 2) during the Mini-OSCEs , with SPIs focusing on the mechanics and MS4s, on the clinical reasoning. SPIs and MS4s also provide formal written summative feedback at the end of each semester. This formal feedback is entered into a rubric housed in an on-line medical education management system at the end of each semester and contributes to their final grade.

## Results

The program uses Kirkpatrick’s levels of appraising medical interventions [20].

### Pre-Clerkship students’ perceptions of the experience (Kirkpatrick level I)

Pre-clerkship students provided their perceptions of the experience through a survey from the GWU department of evaluation and educational research. Ninety-seven percent of the pre-clerkship students strongly agreed or agreed that the physical diagnosis sessions were valuable and included narrative comments:

- ‘I think the physical diagnosis course is medical education at its finest; e.g., having readings that relate to what is being done and reinforcing in class while being taught from the perspective of a 4^th^ year student and a patient.
- ‘I always learned a lot and enjoyed working with my peers, the MS4s and the SPI’.
- ‘The SPIs have good input about practical ways to carry out maneuvers and good tips about nuances that could make a patient uncomfortable’.
- ‘Having an SPI and MS4 that worked together well and were able to give different perspectives on the teaching was highly valuable. The MS4 gave insight as to what the purpose of the teaching was in clinical practice’.
- ‘I wish we had more frequent sessions. This was my favorite part of the Practice of Medicine course and I felt it was over too quickly’.
- ‘This is a good prep for clinical rotations.’
- ‘It’s great to have an opportunity to practice on an SP and each other.’
- ‘It is helpful to learn how to do specific maneuvers with my peers.’

### MS4s and SPIs perceptions of the experience (Kirkpatrick level I) [21]

Of those responding, one hundred percent of both the SPIs (*N*=16 [100%]) and the MS4s (*N*=44 [77%]) reported that their experience as PDX teachers was positive; 91% and 93% respectively of the SPIs and MS4s stated that they had a positive experience working with each other.

### MS4s modifications of attitudes/perceptions (Kirkpatrick IIa) and behavior change (Kirkpatrick III)

Themes that emerged in our qualitative analysis of MS4s’ impressions of what they valued in their experience as teachers [22] included: 1) Implementing adult learning theory: activating learners and providing a safe learning climate; 2) Preparation for teaching: envisioning pertinent clinical applications, predicting pre-clerkship students’ questions, and seeking the answers together; 3) Modeling professionalism; 4) Exceeding expectations: coming early to sessions and leaving late; making certain the pre-session quiz questions were value-added; 5) Feedback: perceiving timely, meaningful, reinforcing and constructive feedback as a priority; 6) Peer counseling: providing advice to pre-clerkship students about study habits, how to best navigate the physical diagnosis course, and career suggestions.

### Pre-Clerkship Students. Behavior in summative assessments (Kirkpatrick III)

Pre-clerkship students participated in a 3-station summative OSCE at the end of the spring semester. To evaluate the effectiveness of our program, we compared pre-clerkship students’ performance on the PE portion of the OSCEs before and after the 2010 initiation of the program. Prior to 2010, faculty physicians working with MS4s taught PDX to pre-clerkship students. Exempting the 2010 transition year, we compared spring OSCE PE results for years 2007 to 2009 vs. 2011 to 2014. The number of students taking the OSCEs ranged from 170-185/ year: 532 students in the pre intervention group and 714 in the post intervention group.

### Statistical methods

Spring exams OSCE results were pooled, weighted by the annual sample sizes, for years 2007 to 2009 and 2011 to 2014. Pooled mean scores across years for the pre- period were compared to pooled mean scores for the post-period using a 2-sample t-test. The GW IRB exempted this study and student consent was obtained to anonymously use their performance data for research.

Mean scores on the physical exam component significantly increased from 83.4 (SD=7.3, *n*=532) preprogram to 89.9 (SD=8.6, *n*=714) post program initiation (mean change = 6.5; 95% CI: 5.6 to 7.4; *p*<0.0001) (Table 3). However, since the shift from faculty to non-faculty instructors coincided with curricular changes, differences in OSCE scores cannot unequivocally be attributed to the innovation.

**Table 3:**
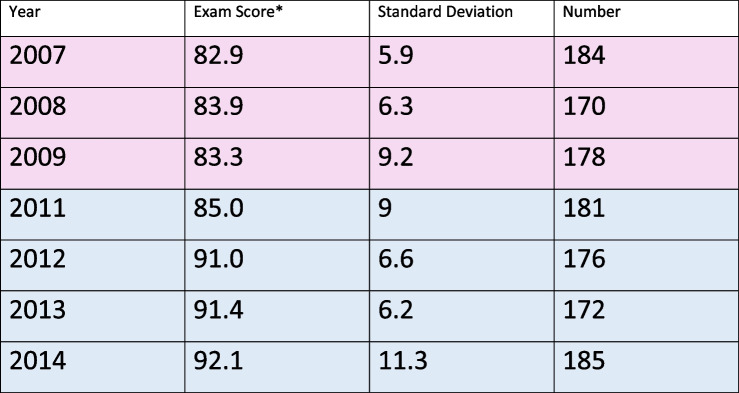
Physical exam scores pre (pink) and post (blue) initiation of MS4-Standardized Patient Instructor Teams

^*^Exam scores are in percent of physical exam items that students performed correctly

Exam checklists contained 15-23 items per case

## Discussion

The SPI-MS4 team instruction model is an innovative approach to teaching basic PE to novice medical students to prepare them for early clinical exposure. It offers an effective alternative by circumventing the barriers associated with faculty involvement. It also offers a value-added feature for both the teaching teams and their pre-clerkship students: they all benefit from co-teaching. Benefits include exposure of pre-clerkship students to different perspectives and to collaborative role modeling [23]. The alternative perspectives inherent in co-teaching create a constructivist environment [10] where these students gain knowledge from dual sources: 1) kinesthetic--building precise technique for PE maneuvers, and 2) synthetic--building diagnostic reasoning. MS4s also benefit from co-teaching, preparing them for their future interdisciplinary work with allied health professionals.

Our model also includes the advantages of near-peer teaching [24]. Pre-clerkship students benefit from the cognitive congruence, safe learning climate, socialization and role modeling; MS4s, from “learning twice”— from their own initial instruction and through teaching others. They also affirm their own professional development through teaching their junior peers, and benefit from faculty-guided opportunities to develop and polish their teaching abilities and exam skills. Further, their teaching experience prepares them to be effective educators when they are residents and faculty by teaching them to use evidenced-based science of teaching and learning practices.

There were lessons learned in implementing this model. First, it is important to recognize the complexities of the interdisciplinary relationship between MS4s and SPIs, with some dyads lacking a clear concept of how to best work together. Clarifying roles, a detailed manual, and the team workshop addressed these problems effectively. Second, detailed training is necessary to optimize team function. While both sets of instructors needed training in how to teach, the SPIs also needed training in how to perform the exam skills that the MS4s already knew. Third, vigilant planning is needed to coordinate with MS4s’ busy schedules, assuring that each physical diagnosis session had a complete team present. Fourth, some faculty/leadership resistance is expected with a new program; citing cost benefits is persuasive.

In summary, the SPI-MS4 physical diagnosis instruction model is a unique and practical curricular innovation through which novice medical students can successfully learn PE skills from carefully trained non-physicians. Since virtually all US and many non-US medical schools use SPs and many have students-as-teachers programs, this model has the potential for wider application.

### Supplementary Information

**Additional file 1.** Form 1: GRPI Planning Form for Co-Teaching Workshop for MS4-Standardized Patient-Instructor Teams.

## Data Availability

The data set for this study is available in the Class Center, GWU, Director, Dr Benjamin Blatt, M.D. All of our data is presented in the study.
