# Supplementary material for: A different approach to teaching pre-clerkship students physical diagnosis: standardized patient instructor-senior medical student teaching teams
Source: BMC Med Educ. 2023 Nov 21;23:887. doi: 10.1186/s12909-023-04782-4 (PMC10664546; doi:10.1186/s12909-023-04782-4)
Supplement: Supplementary file 1 — Additional file 1. Form 1: GRPI Planning Form for Co-Teaching Workshop for MS4-Standardized Patient-Instructor Teams. [file 12909_2023_4782_MOESM1_ESM.docx]

**Form 1:** **GRPI** **Planning Form for Co-Teaching Workshop for MS4-Standardized Patient-Instructor Teams**

(Using Goals, Roles, Process, Interpersonal (GRPI) Team Function Model and Mezirow’s Transformational Learning Theory, MS4s and SPIs work through this form together to define a plan for collaboration in their teaching of physical diagnosis to MS1s)

**GOALS**

Our Goals for our students

a. How prepared do we expect them to be for the session? How will we know?

b. How should they relate to each other during the sessions? How casual/formal should they be?

How should students of different genders relate when examining each other?

c. What do we expect students’ attitudes to be?

Our Goals for ourselves

a. What kind of learning climate do we want to create for our sessions?

b. How do we want our teaching relationship to look to our students?

**ROLES (SPIs and MS4s)**

a. How are our roles similar? How different?

b. What are our roles during Part 1 of the session (mini-quiz, responding to student questions about the physical exam, introducing the cases?)

c. What are our roles during Part 2 of the session (students examine each other)?

d. What are our roles during Part 3 of the session (Cases)?

e. What are our roles when we evaluate students after the session?

**PROCESS/INTERACTIONS**

a. If a problem occurs, are we comfortable using the Mezirow content, premise, process reflection method? (or the GRPI model?)

1. Content: What is the problem?

2. Premise: Why is it happening? (including reflecting on our biases)

3. Process: How can we address it?

b. If a problem occurs, what means will work best for us to discuss it? Talk after the session? Phone? E-Mail?

c. What if a problem occurs during the session? Can either of us call a “time out, ” leave the room and problem solve?

d. How will we evaluate our interaction as a teaching team? Quick review of what went well, what to do differently while doing evaluations?

e. If we differ about something and can’t seem to work it out, are we OK with discussing it with the course directors?
